# Computer Analysis of the Effect of Activation Temperature on the Microporous Structure Development of Activated Carbon Derived from Common Polypody

**DOI:** 10.3390/ma14112951

**Published:** 2021-05-30

**Authors:** Mirosław Kwiatkowski, Jarosław Serafin, Andy M. Booth, Beata Michalkiewicz

**Affiliations:** 1 Department of Fuel Technology, Faculty of Energy and Fuels, AGH University of Science and Technology, al. A. Mickiewicza 30, 30-059 Krakow, Poland; 2 Department of Chemical Engineering and Barcelona Research Center in Multiscale Science and Engineering, Institute of Energy Technologies, Technical University of Catalonia, EEBE Eduard Maristany 10-14, 08019 Barcelona, Spain; jaroslaw.serafin@upc.edu; 3 Department of Climate and Environment, SINTEF Ocean, 7465 Trondheim, Norway; andy.booth@sintef.no; 4 Department of Catalytic and Sorbent Materials Engineering, Faculty of Chemical Technology and Engineering, West Pomeranian University of Technology in Szczecin, ul. Pułaskiego 10, 70-322 Szczecin, Poland; Beata.Michalkiewicz@zut.edu.pl

**Keywords:** biomass, adsorption, microporous structure, chemical activation, activated carbons

## Abstract

This paper presents the results of a computer analysis of the effect of activation process temperature on the development of the microporous structure of activated carbon derived from the leaves of common polypody (*Polypodium vulgare*) via chemical activation with phosphoric acid (H_3_PO_4_) at activation temperatures of 700, 800, and 900 °C. An unconventional approach to porous structure analysis, using the new numerical clustering-based adsorption analysis (LBET) method together with the implemented unique gas state equation, was used in this study. The LBET method is based on unique mathematical models that take into account, in addition to surface heterogeneity, the possibility of molecule clusters branching and the geometric and energy limitations of adsorbate cluster formation. It enabled us to determine a set of parameters comprehensively and reliably describing the porous structure of carbon material on the basis of the determined adsorption isotherm. Porous structure analyses using the LBET method were based on nitrogen (N_2_), carbon dioxide (CO_2_), and methane (CH_4_) adsorption isotherms determined for individual activated carbon. The analyses carried out showed the highest CO_2_ adsorption capacity for activated carbon obtained was at an activation temperature of 900 °C, a value only slightly higher than that obtained for activated carbon prepared at 700 °C, but the values of geometrical parameters determined for these activated carbons showed significant differences. The results of the analyses obtained with the LBET method were also compared with the results of iodine number analysis and the results obtained with the Brunauer–Emmett–Teller (BET), Dubinin–Radushkevich (DR), and quenched solid density functional theory (QSDFT) methods, demonstrating their complementarity.

## 1. Introduction

Activated carbons are well-known for being a very good gas and liquid phase adsorbents for a wide variety of organic and inorganic chemicals, resulting in their used or consideration for a wide range of applications. Importantly, the adsorption capacity and efficiency of activated carbons are significantly influenced by their physicochemical properties, which in turn are dependent on carbon precursor selected and the method of synthesis [1,2,3,4,5,6,7,8,9,10,11]. As the cost of the raw material is important, special attention has been paid to the use of biomass waste as source materials [12,13,14,15,16,17,18,19,20,21]. Industrial production of activated carbons starts with the carbonisation of the raw materials, followed by a physical or chemical activation process, or a combination of these processes. 

Carbonisation is achieved by heating the raw materials without the presence of air or in an inert gas atmosphere [22]. The physical activation process comprises a partial gasification of the carbonisation product at a temperature of 800–1000 °C with oxidising agents such as water steam or carbon dioxide; the use of a mixture of these agents is also possible [23,24]. The use of oxygen at a temperature below 800 °C is possible but is used less frequently.

Chemical activation offers an alternative to the physical activation methods and consists of a one-step process in which the raw material reacts with an activating agent [25,26,27,28]. Chemical activation involves impregnation of the raw material by mixing it with a suitable amount of the selected activating agent, which usually has the form of a concentrated solution. Next the chemically impregnated raw material is carbonised in neutral atmosphere. The product of carbonisation is cooled and rinsed with distilled water or mild acid to remove and recycle any remaining activating agent.

The proper selection of conditions for the process of producing microporous carbonaceous adsorbents and optimising their structure to the specific application conditions requires a precise description of the microporous structure. Physical adsorption of different gases has become the most popular method for characterisation of porous structures. Of the many methods devised to analyse microporous structures on the basis of adsorption isotherms, one of the most popular and commonly used is the BET method. This is derived from the multilayer adsorption theory by Brunauer, Emmet, and Teller and the adsorption process model developed based on that theory [29]. The method is typically used to determine the specific surface area *S_BET_*, but it has known limitations as the assumptions behind the BET model do not take into account the filling of the micropores volume. Despite of its noteworthy accuracy as a comparative approach, the method is not recommended for the analysis of microporous materials.

Considering the pros and cons of the BET theory and the BET multilayer adsorption equation derived therefrom, work was undertaken to develop a new clustering-based adsorption theory based on the fundamentals behind the BET theory and informed by the process of multilayer adsorption on the surface of microporous carbonaceous materials [30,31,32]. The work on the new theory, named uniBET, was based on the principal assumption that it is not possible to unambiguously and credibly determine the distribution of energy on the surface of an adsorbent and the structure of a porous material, yet a mathematical description of the adsorption process should take into account the anticipated geometrical properties of the material and those related to energy on its surface [30,31,32]. That theory served as the basis for developing new adsorption models, as well as a unique procedure for the fast multivariant identification of adsorption systems. Together, these are named the new numerical clustering based adsorption analysis (LBET) method [30,31,32]. The LBET models involve five parameters: *V_hA_* is the volume of the first adsorbed layer; *Q_A_*/*RT* is the dimensionless energy parameter for the first adsorbed layer; *B_C_* is the dimensionless energy parameter for the higher adsorbed layers; *α* is the geometrical parameter of the porous structure determining the height of the adsorbate molecule clusters; *β* is the average number of sites provided by (*n−*1)th adsorbed layer for the *n*th layer, averaged over all adsorbate molecule clusters (*β* = 1 for narrow pores, *β* > 1 for wider ones); *Z_A_* is the effective contact correction factor; *h* is the surface heterogeneity parameter; *σ_e_* is the fitting error dispersion; and *w_id_* is the identification reliability indices, which can be adjusted by fitting of the LBET formula to the adsorption isotherm with a chosen variant of the surface energy distribution function [30,31,32].

To apply the LBET equations for near and supercritical temperatures, an original fluid state model was developed that was fitted with high accuracy compression factor data [33]. The new numerical clustering-based adsorption analysis method with the implemented LBET class models and the fast multivariant procedure of identification of adsorption systems have been presented in detail in earlier papers [30,31,32,33].

## 2. Materials and Methods

The leaves of common polypody (Colombian forests, Bogota, Colombia), a widely distributed type of fern, were used as the material for the production of the activated carbons used in the current study. The raw material was ground into a powder and then subjected to activation with the use of 48% H_3_PO_4_ (Acros Organics, Geel, Belgium) for 3 h. The product of that process was left to dry for 12 h. Next, the material was rinsed with deionised water to remove the remains of H_3_PO_4_, after which, it was carbonised in horizontal furnace by sequential heating (Czylok Company, Jastrzębie-Zdrój, Poland) for 1 h at temperatures of 700, 800, and 900 °C, with nitrogen as the inert gas. These activated carbon samples were labelled as FAC/*T* with *T* indicating the activation temperature. 

Micrographs of the activated carbon samples were obtained using an ultrahigh resolution field emission scanning electron microscope (UHR FE-SEM Hitachi SU8020, Hitachi High-Technologies Corporation, Tokyo, Japan) equipped with an Energy Dispersive X-ray (EDX) detector (Hitachi High-Technologies Corporation, Tokyo, Japan). The samples were mounted on an aluminium stub using carbon conductive adhesive tape. 

Additionally, the iodine number was applied to estimate the porosity of the activated carbons. The iodine number, which is defined as the amount of iodine (mg) adsorbed by 1 g of activated carbon, was calculated on the basis of the sodium thiosulfate volume used in the sample titration. The textural parameters were calculated on the basis of N_2_ adsorption-desorption isotherms at −196 °C; a Quantachrome Autosorb Automated Gas Sorption System QUADRASORB evo (Quatochrome Instruments, Boynton Beach, FL, USA) was utilized. Adsorption of CO_2_ and CH_4_ was performed at a temperature of 25 °C up to pressures of 3.5 and 4.5 MPa, respectively. The measurements were performed using the static volumetric method using the Hiden Isochema IMI–HTP Manometric gas sorption analyser (Hiden Isochema, Warrington, United Kingdom). 

The porous structure of the resultant activated carbons was analysed on the basis of −196 °C nitrogen, carbon dioxide, and methane adsorption isotherms with use of the BET [29], the LBET [30,31,32,33], the Dubinin-Raduskevich (DR) [34], and the quenched solid density functional theory (QSDFT) [35,36] methods. The *P*/*P_0_* range was found for each material on the basis of linearity of BET and DR plots. In the case of surface area, two additional criteria were applied: the positive intercept of the BET plot (C positive) and the term *V*(1−*P*/*P*_0_), which continuously increases with *P*/*P*_0_. The *P*/*P*_0_ ranged from 0.02 to 0.30 for the BET equation and from 0.02 to 0.09 for the DR equation. The total pore volume was calculated on the basis of N_2_ volume adsorbed at a relative pressure, *P*/*P_0_* = 0.98.

## 3. Results and Discussion

**Figure 1:**
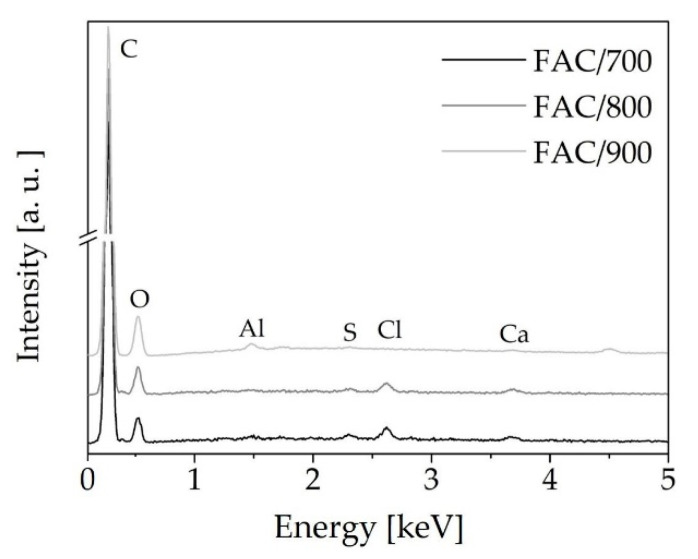
EDX analysis of the activated carbons derived from common polypody leaves at temperatures of 700, 800, and 900 °C.

The chemical composition of activated carbons derived from common polypody leaves was determined using EDX measurements (Figure 1). 

**Figure 2:**
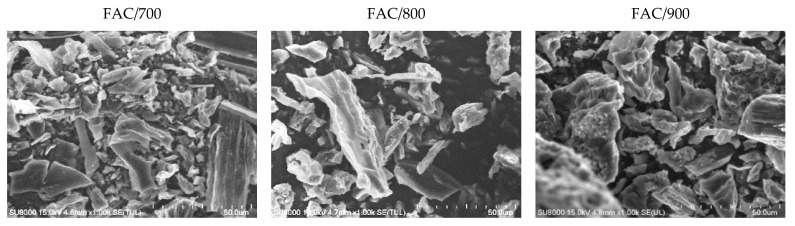
FE-SEM micrographs of the activated carbons derived from the common polypody leaves at activation temperatures of 700, 800, and 900 °C.

The results showed there was no signal from phosphorus, indicating all the phosphoric acid activating agent was removed during the washing step. Only oxygen was present on the surface of the activated carbon, together with trace amounts of S, Cl, and Ca. The presence of S, Cl, and Ca in activated carbons is due to the presence of these elements in common polypody. The traces of alumina were caused by the sample holder and could not be eliminated. The surface topography of the activated carbons produced at activation temperatures of 700, 800, and 900 °C was investigated using FE-SEM micrographs (Figure 2). At all temperatures, the surface of all the activated carbons was fairly smooth. However, a few cavities were observed for the material activated at the temperature 900 °C.

The FE-SEM micrographs show that the particle sizes of the activated carbons produced at different temperatures were similar and ranged from 5 to 50 μm. At 700 °C, smaller particles were produced. The size 5–10 μm was dominant. The average size of activated carbon carbonized at 800 °C was about 10–15 μm and at 900 °C, about 25–30 μm.

Iodine number is often used to estimate the porosity of activated carbons, especially the microporosity. The iodine number values of the activated carbons produced from the common polypody leaves increased as the activation temperature increased, with values of 349, 391, and 433 mg/g being determined for 700, 800, and 900 °C, respectively. These data clearly show that the microporosity of the activated carbons increased with the temperature of activation.

**Figure 3:**
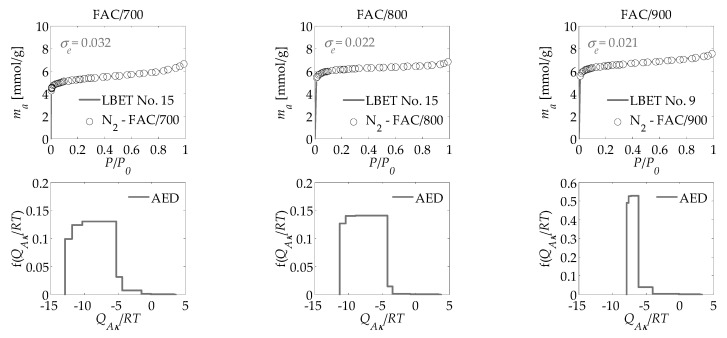
Nitrogen adsorption isotherms for activated carbons derived from the leaves of common polypody and the fast multivariant identification procedure results obtained via the LBET method; where AED is the adsorption energy distribution for the first adsorbed layer.

**Figure 4:**
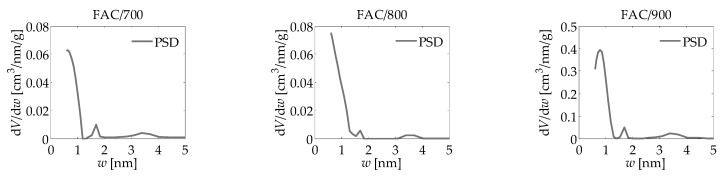
The pore size distributions obtained via the QSDFT method based on nitrogen adsorption isotherms for activated carbons derived from the leaves of common polypody.

**Figure 5:**
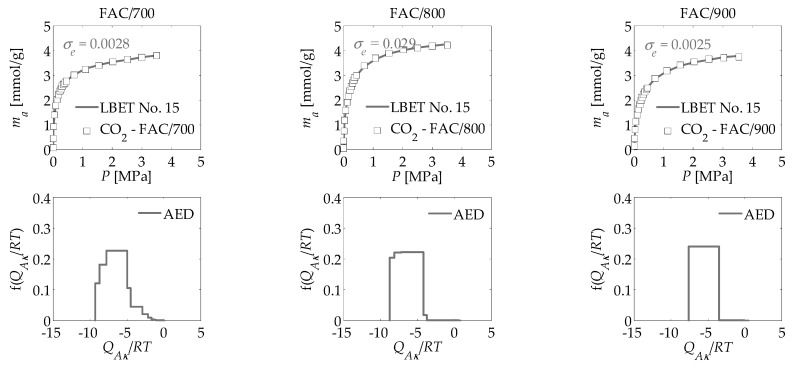
Carbon dioxide adsorption isotherms for activated carbons derived from the leaves of common polypody and the fast multivariant identification procedure results obtained via the LBET method, where AED is the adsorption energy distribution for the first adsorbed layer.

**Figure 6:**
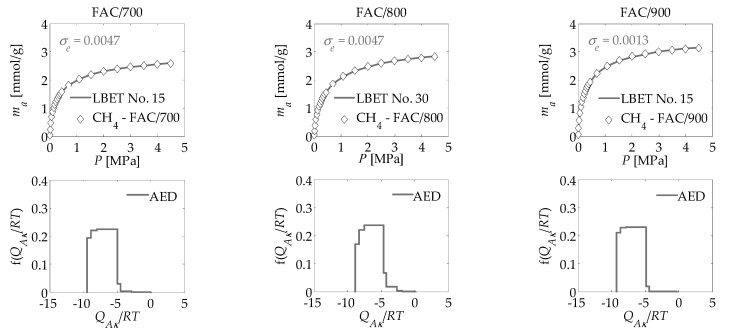
Methane adsorption isotherms for activated carbons derived from the leaves of common polypody and the fast multivariant identification procedure results obtained via the LBET method, where AED is the adsorption energy distribution for the first adsorbed layer.

The main aim of the work was to apply the new numerical clustering-based adsorption analysis (LBET) method to the analysis of the effect of activation process temperature on the development of the microporous structure of activated carbons obtained from the leaves of common polypody via chemical activation with H_3_PO_4_, carried out on the basis of nitrogen, carbon dioxide, and methane adsorption isotherms. The results of the investigation are compiled in Table 1 and Table 2 and presented in Figure 3, Figure 4, Figure 5 and Figure 6.

Analysis of the microporous structure of activated carbon produced at an activation temperature of 700 °C (FAC/700) based on nitrogen adsorption isotherms (Table 1) showed that the material is highly heterogeneous (*h* = 9), while limitations to adsorbate molecule cluster growth result from the competitive development of the neighbouring clusters (LBET model no. 15). Moreover, the obtained parameters of adsorption energy on the first layer (*Q_A_*/*RT*) and the successive layers (*B_C_*) testify to the occurrence of preferential conditions that favour the adsorption of molecules directly on the surface of the adsorbent (*Q_A_*/*RT* = −12.85, *B_C_* = 4.82). The value of the effective contact parameter *Z_A_* (*Z_A_* = 0.536) suggests the existence of micropores in FAC/700, where the dimensions ensure more than one adsorbate molecule is adsorbed. The values of the geometrical parameters *α* and *β* (*α* = 0.25 and *β* = 2.28) obtained by means of the LBET method provide evidence that the micropores of FAC/700 contain low and branched clusters of adsorbate molecules, while the shape of the energy distribution reveals a very broad spectrum of energy of primary adsorption sites on the surface of the analysed material (see Figure 3).

The results generated using the LBET method are also confirmed by the results obtained using the BET, DR, and QSDFT methods (Table 2). However, the specific surface area produced with the BET method differed considerably from the corresponding parameter achieved with the other methods. This empirically highlights the much-discussed issues over the use of the BET method to describe microporous materials. The pore size distribution determined via QSDFT method, based on the nitrogen adsorption isotherms indicates a prevalent proportion of bimodal-structure micropores in the total pore volume, as well as the occurrence of mesopores in the range from 3 to 4 nm (see Figure 4).

The results obtained for the sample of activated carbon produced at an activation temperature of 800 °C (FAC/800) show that the analysed surface is highly heterogeneous (*h* = 9; Table 1) and comparable to FAC/700. However, FAC/800 exhibits higher values for the parameters *V_hA_* (0.198 cm^3^/g; volume of the first adsorbed layer) and *B_C_* (6.91; energy parameter for the higher adsorbed layers) compared with FAC/700, where *V_hA_* and *B_C_* are 0.167 cm^3^/g and 4.82, respectively. Furthermore, the research carried out with the LBET method revealed that the clusters of nitrogen molecules developed in the micropores of FAC/800 are low and significantly branched (*α* = 0.10 and *β* = 3.04), i.e., adsorption to a single molecule involves two or more molecules of the successive layers.

Analogous to FAC/700, the shape of the FAC/800 surface energy distribution indicates the occurrence of a broad range of energy of primary adsorption sites (Figure 3), while the shape of the PSD is also similar to that for FAC/700. Higher values of the parameters *S_BET_*, *S_DR_*, *S_QSDFT_*, and *V_DR_* (i.e., *S_BET_* = 423.5 m^2^/g, *S_DR_* = 610.7 m^2^/g, *S_QSDFT_* = 643.9 cm^3^/g, and *V_DR_* = 0.212 cm^3^/g) were obtained for FAC/800 compared with FAC/700 (Table 2), which shows that the development of the porous structure of the analysed material advances in parallel with an increase in temperature.

The results obtained for the activated carbon prepared at an activation temperature of 900 °C (FAC/900) show that the material in question is heterogeneous (*h* = 5), although less so than FAC/700 and FAC/800 (Table 1). The material is also characterised by having the highest *V_hA_* value among all three of FAC samples (*V_hA_* = 0.211 cm^3^/g), but the lowest values of the energy parameters *Q_A_*/*RT* and *B_C_* (i.e., −7.85 and 3.35, respectively). The nitrogen molecule clusters developed in the micropores of the activated carbon FAC/900 are low and branched, as evidenced by the value of the geometrical parameters *α* and *β* (*α* = 0.15 and *β* = 1.76). The shape of the adsorption energy distribution on the first adsorbed layer points to the occurrence of a narrower range of adsorption energy compared with the samples analysed before (Figure 3). Furthermore, the analyses performed by means of the BET, the DR, and the DFT methods revealed that FAC/900 has the largest specific surface area and the highest volume of pores, including micropores (Table 2). Very good fitting was obtained for all the analysed isotherms of nitrogen adsorption, as evidenced by the values of fitting error dispersion *σ_e_*, while the values of the identifiability indicator *w_id_* point to a good identifiability of the adsorption systems subject to the analysis (Table 1).

Analysis of the porous structure of the activated carbons based on carbon dioxide adsorption isotherms produced very interesting results compared to those obtained for the nitrogen adsorption isotherms (Table 1). All three activated carbon materials are strongly heterogeneous, which is in line with the results obtained for FAC/700 and FAC/800 on the basis of nitrogen adsorption isotherms, but an increase relative to the value for FAC/900. For all three activated carbon materials, analyses conducted on the basis of CO_2_ adsorption isotherms yielded higher values for *V_hA_*, *Q_A_*/*RT*, *Z_A_* and *α* (with the exception of FAC/900), but lower values for *B_C_* and *β*, when compared with data obtained on the basis of the nitrogen adsorption isotherms (Table 1). The shape of the adsorption energy distribution on the first adsorbed layer clearly points to the occurrence of a broad range of energy of primary adsorption sites for all three activated carbon (Figure 5). This is comparable to the results observed for FAC/800 produced using the nitrogen isotherm (Figure 3). 

The results produced on the basis of carbon dioxide adsorption isotherms provide evidence that the size of the micropores on the surface of all three activated carbons is comparable to the size of CO_2_ molecules. Furthermore, the adsorbate molecule clusters developed in these micropores are medium-sized and stack-like in shape. Equally interesting results were obtained from the analysis of the porous structure of the activated carbon based on methane adsorption isotherms. For all three activated carbon materials, the calculated *V_hA_* values were lower for the methane adsorption isotherms than for both the nitrogen and CO_2_ isotherms (Table 1). The energy parameters *Q_A_*/*RT* and *B_C_* obtained for the FAC/700 and FAC/800 methane isotherms are similar to those determined for the carbon dioxide adsorption isotherms. However, this trend changes for FAC/900, where *Q_A_*/*RT* is higher and *B_C_* is markedly lower for the methane isotherm than the CO_2_ isotherm. The *Z_A_* values derived on the basis of methane adsorption isotherms are the highest for all the activated carbon materials compared with those based on nitrogen and CO_2_ adsorption isotherms, although they are similar in most cases to the values for the CO_2_ isotherms. Conversely, the value determined for the height of the methane molecule clusters (*α)* developed in the pores of the successively analysed samples is consistently smaller than for the nitrogen and CO_2_ molecule clusters. Moreover, an LBET-based analysis of carbon dioxide and methane adsorption isotherms produced lower values of the adsorbate molecule cluster width parameters.

The shape of the adsorption energy distribution on the first adsorbed layer clearly points to the occurrence of a broad range of energy of primary adsorption sites for all three activated carbon materials (Figure 6). This is comparable to the results observed for FAC/800 produced using the nitrogen isotherm (Figure 3) and to the results for all three activated carbon materials produced using the CO_2_ isotherms (Figure 5). Based on our analyses, a correspondence between the parameters of the microporous structure and the iodine number can be obtained. The iodine number values of activated carbons produced from common polypody leaves correspond well with values for the volume of the first adsorbed layer (*V_hA_*); values for the specific surface determined by the BET, DR, and DFT (*S_BET_, S_DR_,* and *S_QSDFT_*) and values for the total pore volume (*V_Total_*, *V_DR_*, and *V_QSDFT_*).

## 4. Conclusions

The main aim of this work was to apply the new numerical clustering based adsorption analysis (LBET) method to the analysis of the effect of activation process temperature on the development of the microporous structure of activated carbon derived from the leaves of common polypody via activation with phosphoric acid. As the findings showed, the LBET method offers a substantial advantage compared with the use of only the most popular equations of adsorption isotherms. The structural parameters obtained from the LBET method on the basis of using different adsorbates depart significantly from one another, meaning that the properties of the particular adsorption systems are also dissimilar. 

The conducted research also indicates that it is particularly advantageous to employ the different gaseous adsorbates to gain a more comprehensive understanding of the adsorption process and capacity of a material. This is particularly relevant when the materials in question are being developed for application as adsorbents. Moreover, the research described herein provided evidence that an analysis of the structure of the adsorption isotherms of different adsorbates, in this case nitrogen, carbon dioxide, and methane, enables the gathering of comprehensive information on the adsorptive properties of the analysed materials. Interestingly, analysis of the carbon dioxide and methane adsorption isotherms for the specific active carbon materials investigated in the current study yielded comparable results when carried out using the LBET method. However, these results departed significantly from the values determined for the same activated carbon materials on the basis of nitrogen adsorption isotherms. 

The LBET method used in the current study is considered fit for analysis of the porous structure of adsorbents on the basis of the adsorption isotherms of the substances for the adsorption process of which these materials are designated. As a result, the adsorptive properties of activated carbons can be established with accuracy and, subsequently, the methods and process conditions of their production can be selected with clockwork precision, taking into account the economic aspect. 

## Figures and Tables

**Table 1:** Compilation of the results from the analysis of the activated carbons derived from the leaves of common polypody porous structure via the LBET method based on nitrogen, carbon dioxide, and methane adsorption isotherms; LBET No. is the number of the best fitted LBET class model; *V_hA_* is the volume of the first adsorbed layer; *Q_A_*/*RT* is the energy parameter for the first adsorbed layer; *B_C_* is the energy parameter for the higher adsorbed layers; *α* is the parameter of the porous structure determining the height of the adsorbate molecules clusters; *β* is the parameter of the porous structure determining the width of the adsorbate molecules clusters; *Z_A_* is the effective contact correction factor; *h* is the heterogeneity parameter; *σ_e_* is the error dispersion of the best fitted model; *w_id_* is the identifiability index.

Adsorbate	LBET No.	*V_hA_* [cm^3^/g]	*Q_A_*/*RT*	*B_C_*	*Z_A_*	*h*	*α*	*β*	*σ_e_*	*w_id_*
FAC/700										
N_2_	15	0.167	−12.85	4.82	0.536	9	0.25	2.28	0.032	0.40
CO_2_	15	0.241	−9.26	0.64	0.807	9	0.48	1.14	0.003	0.76
CH_4_	15	0.154	−9.45	0.96	0.904	9	0.13	1.00	0.005	0.64
FAC/800										
N_2_	15	0.198	−11.29	6.91	0.496	9	0.10	3.04	0.022	0.24
CO_2_	15	0.220	−8.73	1.51	0.818	9	0.29	1.00	0.029	0.34
CH_4_	30	0.179	−8.87	0.92	0.854	9	0.08	1.00	0.012	0.36
FAC/900										
N_2_	9	0.211	−7.85	3.35	0.406	5	0.15	1.76	0.021	0.51
CO_2_	15	0.243	−7.59	2.53	0.704	9	0.08	1.00	0.025	0.30
CH_4_	15	0.190	−9.26	0.92	0.884	9	0.01	1.00	0.013	0.43

**Table 2:** Compilation of the results from the analysis of the activated carbons porous structure via the BET, DR, and QSDFT methods based on nitrogen adsorption isotherms; *S_BET_* is the specific surface area determined by the BET method, *S_DR_* is the specific surface area determined by the DR method, *S_QSDFT_* is the specific surface area determined by the QSDFT method, *V_Total_* is the total pores volume determined at a relative pressure *P/P_0_* = 0.98, *V_DR_* is the micropores volume determined by the DR method, *V_QSDFT_* is the micropores volume determined by the QSDFT method, *D_DR_* is the peak pore size determined by the DR method, and *D_QSDFT_* is the peak pore size determined by the QSDFT method.

Sample	*S_BET_* [m^2^/g]	*S_DR_* [m^2^/g]	*S_QSDFT_* [m^2^/g]	*V_Total_* [cm^3^/g]	*V_DR_* [cm^3^/g]	*V_QSDFT_* [cm^3^/g]	*D_DR_* [nm]	*D_QSDFT_* [nm]
FAC/700	366.7	519.9	615.7	0.230	0.185	0.212	1.179	1.556
FAC/800	423.5	610.7	643.9	0.236	0.212	0.217	1.204	1.786
FAC/900	448.5	644.0	664.3	0.262	0.229	0.241	1.235	1.725

## Data Availability

The data presented in this work are available upon request.
